# Microfluidics-assisted fabrication of natural killer cell-laden microgel enhances the therapeutic efficacy for tumor immunotherapy

**DOI:** 10.1016/j.mtbio.2024.101055

**Published:** 2024-04-17

**Authors:** Dongjin Lee, Seok Min Kim, Dahong Kim, Seung Yeop Baek, Seon Ju Yeo, Jae Jong Lee, Chaenyung Cha, Su A Park, Tae-Don Kim

**Affiliations:** a Immunotherapy Convergence Research Center, Korea Research Institute of Bioscience and Biotechnology (KRIBB), Daejeon, 34141, Republic of Korea; b Nano-Convergence Manufacturing Research Division, Korea Institute of Machinery and Materials (KIMM), Daejeon, 34103, Republic of Korea; c Department of Functional Genomics, KRIBB School of Bioscience, Korea University of Science and Technology (UST), Daejeon, 34113, Republic of Korea; d Department of Applied Bioengineering, Seoul National University, Seoul, 08826, Republic of Korea; e School of Materials Science and Engineering, Ulsan National Institute of Science and Technology, Ulsan 44919, Republic of Korea; f Department of Biomedical Engineering, School of Life Sciences, Ulsan National Institute of Science and Technology, Ulsan, 44919, Republic of Korea

**Keywords:** Double flow-focusing microfluidics, NK92 cell encapsulation, Microgel mechanics, Microgel degradation, Cancer immunotherapy

## Abstract

Recently, interest in cancer immunotherapy has increased over traditional anti-cancer therapies such as chemotherapy or targeted therapy. Natural killer (NK) cells are part of the immune cell family and essential to tumor immunotherapy as they detect and kill cancer cells. However, the disadvantage of NK cells is that cell culture is difficult. In this study, porous microgels have been fabricated using microfluidic channels to effectively culture NK cells. Microgel fabrication using microfluidics can be mass-produced in a short time and can be made in a uniform size. Microgels consist of photo cross-linkable polymers such as methacrylic gelatin (GelMa) and can be regulated via controlled GelMa concentrations. NK92 cell-laden three-dimensional (3D) microgels increase mRNA expression levels, NK92 cell proliferation, cytokine release, and anti-tumor efficacy, compared with two-dimensional (2D) cultures. In addition, the study confirms that 3D-cultured NK92 cells enhance anti-tumor effects compared with enhancement by 2D-cultured NK92 cells in the K562 leukemia mouse model. Microgels containing healthy NK cells are designed to completely degrade after 5 days allowing NK cells to be released to achieve cell-to-cell interaction with cancer cells. Overall, this microgel system provides a new cell culture platform for the effective culturing of NK cells and a new strategy for developing immune cell therapy.

## Highlights

• Porous microgels have been fabricated using microfluidic channels to effectively culture NK cells.
• NK92 cell-laden three-dimensional (3D) microgels increase cell proliferation, cytokine release, and anti-tumor efficacy.
• 3D-cultured NK92 cells enhance anti-tumor effects in the K562 leukemia mouse model.

## Graphical abstract

**Figure:**
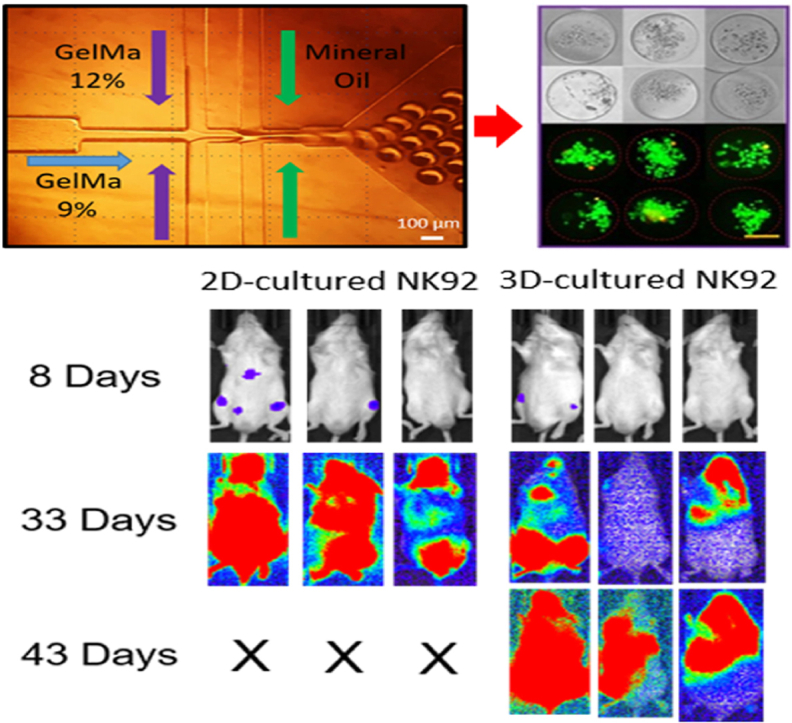
Microfluidics-assisted fabrication of natural killer cell-laden microgel for tumor immunotherapy

## 1 Introduction

Tumors are a global human health issue, and various anti-cancer therapies such as surgical-, chemo-, radiation-, and immunotherapy have been used to treat it [1]. Among these therapies, the therapeutic strategy using immune cells capable of recognizing and killing tumor cells is of interest as a next-generation anti-cancer therapy [2,3]. In recent years, many studies on the chimeric antigen receptor (CAR) have focused on T cells [4]. However, it takes a long time to modify donated T cells to proliferate them, and it is too expensive to use. CAR-T therapy is generally limited to blood tumors [5]. Additionally, further clinical trials may be limited because cytokine release syndrome (CRS) or other side effects may occur from the off-target [6].

Unlike T cells, Natural killer (NK) cells can distinguish tumor sites in an antigen-independent manner [[7], [8], [9], [10], [11]], and have no memory function or clonal expansion, resulting in fewer side effects like CRS. Since allogeneic cell transfer therapy is possible it offers the advantage of being available as an off-the-shelf product [12]. Through preclinical and clinical studies, the safety and efficacy of allotransplantable NK cells for assorted types of hematological and solid cancers have been indicated, and various clinical trials are currently in progress [6]. However, the disadvantages of NK cells include a low proliferation rate and the inability to be cultured for a long period of time [13]. In addition, maintaining the cytotoxic activity of NK cells is important in cell therapy, but it is difficult to maintain it in general two-dimensional cultures and cell proliferation is not easy in in-vivo systems [14]. Previous studies have reported that NK cell encapsulated three-dimensional (3D) cultures show an improved anticancer effect. For example, after fabricating a scaffold with hyaluronic acid, NK cells were cultured in the scaffold and used as a method to prevent cancer metastasis when transplanted to the cancer site [15]. Microgels were fabricated to contain immune cells in an emulsion method, and solid cancer disease models were demonstrated through in-vitro experiments [16]. However, the NK cell-laden microgel researches for anticancer immunotherapy has not yet been studies. Furthermore, an in-vivo experiment was performed in which cancer cells were killed through intravenous injection of a NK cell-laden microgels which would be degraded.

Liquid droplets with a monodisperse size and structure can be produced using double flow-focusing microfluidics, which has lately attracted attention in the field of biomedical engineering because it offers a high yield and biocompatibility [[17], [18], [19]]. Double flow-focusing microfluidics was used to create cell-containing photo-crosslinkable methacrylic gelatin (GelMa) monodisperse droplets, while cell-containing microgels with high viability were produced through cross-linking. There have been several previous studies that have generated microgels containing cells using microfluidic chips [[20], [21], [22]]. Microgels have the advantage of being able to easily control the modulus of elasticity by controlling the concentration of biopolymers. It can be applied efficiently to produce monodisperse droplets containing a large number of biomolecules and enable high-throughput examination and injectability. The generated droplets can be transformed into microgels through crosslinking and applied in multiple ways depending on the characteristics of the material [20,[23], [24], [25], [26]]. However, no study has reported the use of a uniform microgel containing NK92 cells using a microfluidic chip yet.

GelMa is one of the materials that can be applied to tissue engineering scaffolds [[27], [28], [29], [30]], drug included carriers [31], and electrochemical sensors [32,33] because it is highly biocompatible and its physical properties can be controlled by regulating its concentration. GelMa is composed of arginine, glycine, and aspartic acid, which are present in large amounts in the molecule, and is designed to be decomposed by the matrix metalloproteinase [[34], [35], [36]]. There is also growing interest in 3D cell culture models to enable more multiple and physiologically appropriate biological assays compared to traditional two-dimensional (2D) monolayer cell cultures [[37], [38], [39], [40]].

In this study, we encapsulated NK92 cells in the microgels (3D) using a microfluidic chip, and their activity was compared with that of the 2D cultured NK92 cells. We observed that NK92 cell-laden microgels increased cell proliferation, mRNA expression levels, cytokine release, and anti-tumor efficacy, compared to the 2D cultures. In addition, we confirmed that the 3D cultured NK92 cells had enhanced anti-tumor effects than the 2D cultured NK92 cells in the K562 leukemia mouse model. The microgel containing NK cells which maintained their activity was designed to be completely degraded after 5 days in order to allow cell to cell interaction with cancer cells. We have shown that microgels have no diffusion limit because of their size (approximately 100 μm) and can be injected directly into the vein using a syringe instead of surgery. These results suggest that microgels containing NK92 cells can be used to provide a new platform for anticancer immunotherapy.

## 2 Materials and methods

### 2.1 Synthesis of methacrylic gelatin (GelMa)

Gelatin (20 g) was dissolved in distilled dimethyl sulfoxide (DMSO) at 50 °C. After the solution was fully mixed, 4-dimethylaminopyridine (2 g, Sigma Aldrich) and 4-methoxyphenol (0.2 g, Sigma Aldrich) were added in the DMSO solution (200 mL) at 50 °C. Glycidyl methacrylate (8 mL, Sigma Aldrich) was very slowly added to the mixed solution and the reaction was maintained for 2 d at 50 °C under dry nitrogen atmosphere. The mixed solution was dialyzed completely against deionized water and lyophilized to get GelMa. The chemical structure of GelMa was analyzed using ^1^H NMR spectroscopy (400-MR DD2, Agilent) (Fig. S1).

### 2.2 Microfluidics-assisted fabrication of NK92 cells encapsulated microgel

**Fig. 1:**
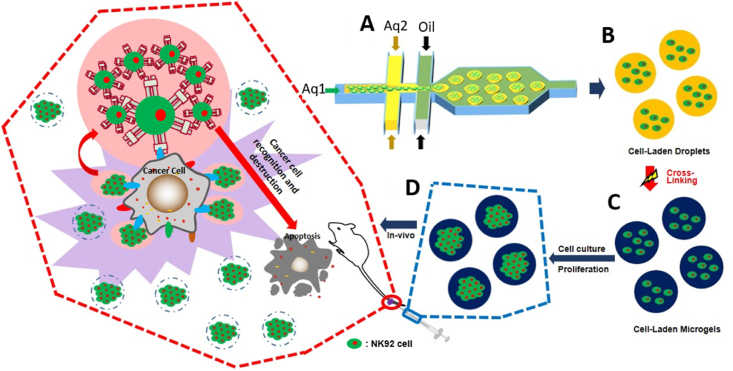
The schematic of overall experiments. (a) Generation of cell-laden microgel using microfluidic chip (b) NK92 Cell-laden droplet formation as precursor solution (c) Cell laden microgel formation using UV light (d) Injection on the mice using cell encapsulated microgel for cell cultured up to 5 days.

The microfluidic chip-based fabrication of cell laden microgels are shown in Fig. S2. Using a geometric microfluidic chip, the GelMa solution could flow in two kinds of channel, such as Aq1 and Aq2 (Fig. 1A). We used four sets of Aq1 and Aq2 concentrations, including 7 % and 10 % (w/v), 9 % and 12 % (w/v), 11 % and 14 % (w/v), and 13 % and 16 % (w/v), respectively. Aq1 included cells and was lower in concentration than Aq2, which contained GelMa. This protected the live cells from the oil, which included surfactants, based on the geometry of the chip design. The flow rate of Aq1 and Aq2 was 135 μL h^−1^, while the flow rate of the oil phase was 900 μL h^−1^. The droplet size was around 100 μm diameter. Initially, the generated cell encapsulated droplets had a monodisperse shape, but they were changed from droplet to microgel through UV exposure (intensity: 0.26 W; distance: 3 cm, emission filter: 250–450 nm; S1500, Omnicure) for crosslinking for 2 min. NK92 cells were mixed in Aq1 at a concentration of 1 × 10^7^ cells mL^−1^. Aq1 and Aq2 included 0.2 % (w/v) irgacure2959 making them photo-initiators. Mineral oil was mixed with 20 % (v/v) Span80 (Sigma Aldrich), a surfactant, to maintain the surface tension to prevent droplet fusion. The fluid flow was controlled by a syringe pump (Legato100, KD Scientific). The obtained microgels were cleaned with cell culture media three times to remove residual mineral oil phase containing the surfactant from the microgel surface. NK92 cell encapsulated microgels were cultured in fresh media containing alpha minimum essential medium (MEM), EtOH, and sulfur at 37 °C and 5 % CO_2_.

### 2.3 Physical property of microgels

GelMa prepolymer materials at different conditions, from 8.4 % to 14.4 % (w/v), with 0.2 % (w/v) irgacure2959 in phosphate buffered saline (PBS, pH 7.4) are shown in Fig. S3. The GelMas with different concentrations were placed on petridishes with the gels of each concentration separated by 1.5 mm space and were exposed to UV radiation for 2 min (intensity: 0.26 W cm^−2^; emission filter: 250–450 nm; distance: 3 cm; S1500, Omnicure) to cure the hydrogel. As it is difficult to measure the elastic modulus of the microgels directly, we fabricated the disk formation of the hydrogel, which was punched out (8 mm diameter) and dipped in PBS 24 h before measuring the elastic modulus. The mechanical properties of GelMa hydrogels were estimated by measuring the elastic moduli with uniaxial compression tests (Model 3343, Instron) [41,42]. Each disk formation of hydrogel was compressed at a fixed rate (1 mm min^−1^) and a stress-strain curve was developed. The strain-stress curve of the elastic modulus was calculated at the initial 10 % of strain. After irradiating with UV for 1 min, it did not change into a gel, but after irradiating with UV for 2 min, it turned into a gel, which is considered sufficient time. The swelling ratio was also confirmed with 8 mm bulk hydrogel. They are soaked in PBS (1 × ) for one day and weighed, then frozen for one day and initially weighed by lyophilization. The swelling ratio was calculated using the difference.

### 2.4 In vitro evaluation

*Cell culture*: Alpha MEM supplemented with 12.5 % fetal bovine serum (FBS), 12.5 % horse serum, three other components (0.2 mM inositol, 0.1 mM 2-mercaptoethanol, 0.02 mM folic acid), 200 U mL^−1^ recombinant IL-2 (Peprotech, Cat. 200-02), and 1 % penicillin/streptomycin (Thermo Fisher) was used to culture the NK92 cells (human natural killer cell line, ATCC CRL-2407TM), while RPMI 1640 medium containing 10 % FBS and 1 % penicillin/streptomycin was used to culture the K562 cell line (human leukemia cell line, ATCC CCL-86TM). Both cell lines were obtained from the American Type Culture Collection (Manassas, VA, USA) and were maintained at 37 °C and 5 % CO_2_.

### 2.5 NK92 cell viability and proliferation

The viability of the cells encapsulated in the microgels was evaluated using the LIVE/DEAD Cell Viability Assay kit (Thermo Fisher), by following the manufacturer's instructions. To prepare the staining solution, calcein-AM (5 μL) and ethidium homodimer-1 (20 μL) were added to PBS (1 × ; 10 mL), which fluorescently labels the living (green) and dead (red) cells. The cell-laden microgels were treated with the staining solution (1 mL) for 1 h, followed by washing with PBS (1 × ). The fluorescence intensity of the stained NK92 cells was observed using a fluorescence microscope (Eclipse Ti; Nikon, Japan). The viability of the NK92 cells was observed every 1, 3, 5, and 7 d by counting the number of living and dead cells. The viability was reported as the percentage of live cells among the total number of cells. To determine the proliferation rate (*k*_P_) of the cell-laden microgel, the number of live cells was counted at various time points, and the normalized number of viable cells (*N*_t_/*N*_0_) was plotted against time (*t*) using the following power-law equation:

(1) $$\frac{N_{t}}{N_{0}}=2^{k_{p}\cdot t}$$

where, the variable *N*_t_ represents the number of viable cells at time *t*, while *N*_0_ represents the initial number of viable cells at *t* = 0 [43,44].

### 2.6 Calcein AM-based cytotoxicity assay

To explore the functionality of the encapsulated NK92 cell microgels, the photo-crosslinked microgels were degraded by collagenase D. The microgels were incubated for 20 min to allow complete degradation and facilitate the interaction between the NK92 cells and target cells. The resulting 3D cultured cells were collected via centrifugation. To perform a cytotoxicity test, the target cells (K562) were stained with calcein-AM (Invitrogen, USA) for 1 h at 37 °C. Next, the labeled target cells (1 × 10^4^/100 μL) and NK92 cells were placed in 96-well round-bottom plates at effector-to-target (E/T) ratios of 4:1, 2:1, 1:1, and 0.5:1 for 2 h. To determine the maximum value of calcein, which corresponds to 100 % killing of the cancer cells, 4 % Triton X-100 was used. The percentage of cytotoxicity was calculated using the following formula: (sample release - background value)/(maximum release - background value) × 100.

### 2.7 Flow cytometry assay

After exposure to collagenase, the NK92 cells were washed with fluorescence-activated cell sorting (FACS) buffer (PBS supplemented with 0.1 % FBS and 0.4 % EDTA) and centrifuged at 100×*g* to obtain cell pellets. The pellets were stained with NK92 cell-specific antibodies for 30 min and analyzed using FACS. The analysis results are presented in Fig. S5.

### 2.8 Real-time PCR (RNA analysis)

To extract the total RNA, Tri-Solution (BSK-Bio Science, Korea) was used in accordance with the manufacturer's protocol. The extracted RNA was reverse-transcribed using a complementary DNA synthesis kit (Toyobo, Japan), and real-time PCR was performed using SFCgreen (SFC, Korea), on the Thermal Cycler Dice Real-Time System III (Takara, Japan). To ensure reliable quantification, all measurements were normalized to the expression of the glyceraldehyde-3-phosphate dehydrogenase (GAPDH) genes.

### 2.9 Cytokine enzyme-linked immunosorbent assay (ELISA) analysis

The effect of cytokine release on the target cells (K562) was measured using the enzyme-linked immunosorbent assay (ELISA) method. The supernatant collected from the co-culture of the tumor cell media and NK cells was used to investigate the concentration of IFN-gamma and TNF-alpha using Invitrogen's ELISA kit (Human IFN-gamma uncoated ELISA, Human TNF-alpha uncoated ELISA), as per the manufacturer's instructions.

### 2.10 Degradation of microgel

GelMa concentrations of 6 % and 9 % were used so that the microgels containing NK cells could be degraded naturally through cell metabolism to react with tumor cells. The UV irradiation time was 2 min, and the intensity was 0.2 W cm^−2^. After washing the oil layer, the experiment was performed under conditions in which all microgels were decomposed within 7 d because these microgels could not be cross-linked completely owing to the use of low GelMa concentrations.

#### 2.10.1 Scanning Electron Microscopy (SEM)

SEM (Model S-4800, Hitachi, Tokyo, Japan) was employed to compare the pore morphology and size trend depending on the GelMa stiffness. After fabricating microgels with four different concentrations, each microgel type was placed on the carbon tape attached on the sample holder. Platinum coating was performed for a minute and each sample was scanned at 5 kV HV and 1200 × magnitude.

### 2.11 Animals and Experimental design

*Mice*: Mice of age 8–12 wk were acquired from SAERONBIO Inc. (Korea). All animal experiments were conducted in compliance with the guidelines of the Institutional Animal Care and Use Committee (IACUC) of the Korea Research Institute of Bioscience and Biotechnology (KRIBB). Mice were purchased form saeronbio (Korea) and used between the ages of 8–12 weeks.

### 2.12 K562 leukemia mouse model

Single cell suspension of 1 × 10^6^ luciferase expressing K562 cells in PBS (200 μL) was injected intravenously into the tail vein of NOD/Shi-scid IL-2Rγnull (NOG) mice on day 0. Moreover, 2D or 3D cultured NK92 cells were intravenously transfused three times (days 0, 4, and 7). After final injection, the condition, body weight, and survival rate of the mice were checked every two days.

### 2.13 Bioluminescence imaging (BLI)

BLI was performed using the IVIS Imaging System (Caliper Life Sciences, USA) every 3–4 d. Mice were anesthetized with 1∼3 % isoflurane, and D-luciferin (PerkinElemer, USA) was intraperitoneally injected into the mice at a concentration of 150 mg per kg bodyweight. BLI was analyzed using the Living Image software (PerkinElemer) and the total flux was calculated as photons per second.

### 2.14 Statistical analyses

Triplicates of *in vitro* samples were tested and each experiment was independently conducted at least twice. Statistical significance was evaluated using *t*-test, two-way ANOVA, or Mantel-Cox test, using GraphPad Prism (version 7.0).

## 3 Results

### 3.1 Microfluidic fabrication of NK92 cell encapsulated microgels

The production of cell-encapsulated microgels was performed using a microfluidic chip with a double flow-focusing geometric design (Fig. 1A). A droplet containing the cells was prepared using a microfluidic chip and was transformed into a microgel through photo-crosslinking. (Fig. 1A, B, and 1C; Fig. S2). The microfluidic chip consisted of an aqueous layer (Aq1 and Aq2), an oil layer, three inlets, and one outlet. In the case of Aq1, a droplet core containing cells was created, and Aq2 acted as a shield from the toxicity of surfactant in the oil layer, helping the cells maintain a high survival rate [20,45]. GelMa, a biocompatible polymer, was used and the concentration of Aq2 was designed to be higher than that of Aq1, thereby limiting the movement of cells included in Aq1 to the outside of the droplet. It was also confirmed that the cells were well maintained in the center of the microgel even after UV cross-linking.

### 3.2 Characterization of microgel properties

**Fig. 2:**
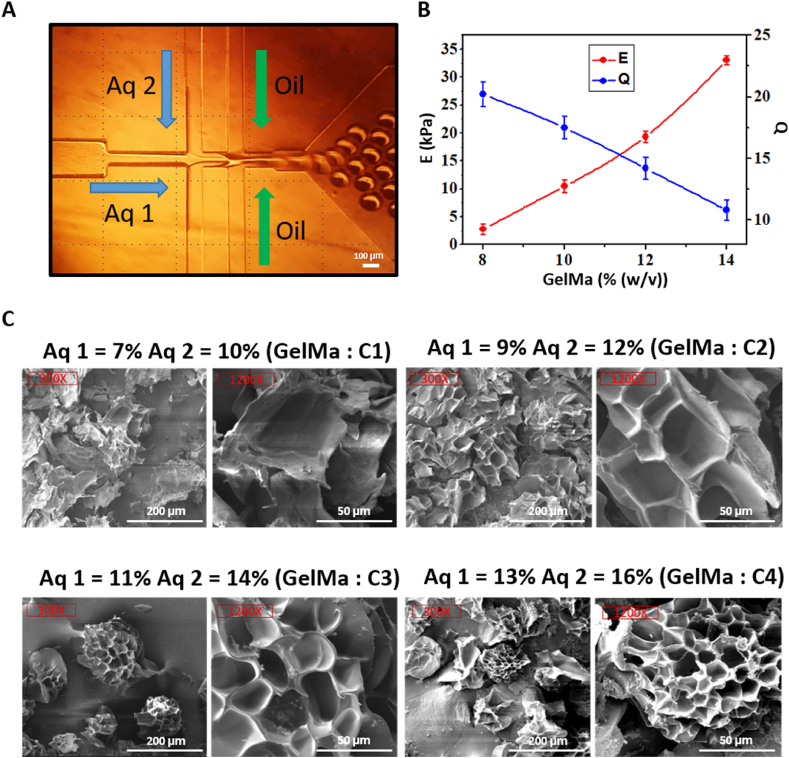
(a) Image of NK92 cell-laden microgel fabrication using a microfluidic chip. (b) Mechanical property (Red) and swelling ratio (Blue) analyses, based on different GelMa concentrations. (c) SEM images of various GelMa concentrations when exposed to same intensity of UV irradiation at 0.26 W cm^−2^ for 2 min. (For interpretation of the references to color in this figure legend, the reader is referred to the Web version of this article.)

Fig. 2A shows the image of a cell-laden microgel created using a microfluidic chip, while Movie S1 shows the process involved in the development of the microgel. We found that it was possible to easily regulate the mechanical properties of the microgels by changing the concentration of GelMa. Four different sets of Aq1 and Aq2 concentrations were assessed: 7 % and 10 % (C1), 9 % and 12 % (C2), 11 % and 14 % (C3), and 13 % and 16 % (C4). Since the size of the microgels were too small to directly measure their elastic modulus, a disk formation of bulk GelMa hydrogel of the same concentration was used for the assessment. Their elastic moduli were measured using uniaxial compression. Stiffness of NK92 cell-laden microgels were controlled by varying GelMa concentration from 8.4 % to 14.4 % (Fig. 2B). This study measured the elastic moduli at concentrations of C1, C2, C3, and C4, which were determined to be 3, 10, 17.5, and 33 kPa, respectively (Fig. 2B, Fig. S3). As the polymer concentration increased, the stiffness increased as well, due to increasing cross-linking density. In contrast, the swelling ratio (Q) shows a reverse phenomenon because soft hydrogels can contain a lot of water, compared to hard hydrogels (Fig. 2B). Based on our observation of the pore size from C1 to C4, using SEM, the weaker the gel strength the larger the pore size, and vice versa (Fig. 2C). This is consistent with the findings of previous reports [43].

The following are the Supplementary data related to this article:

### 3.3 Optimization of NK92 cell-laden microgels

#### 3.3.1 Screening of NK92 cell density

**Fig. 3:**
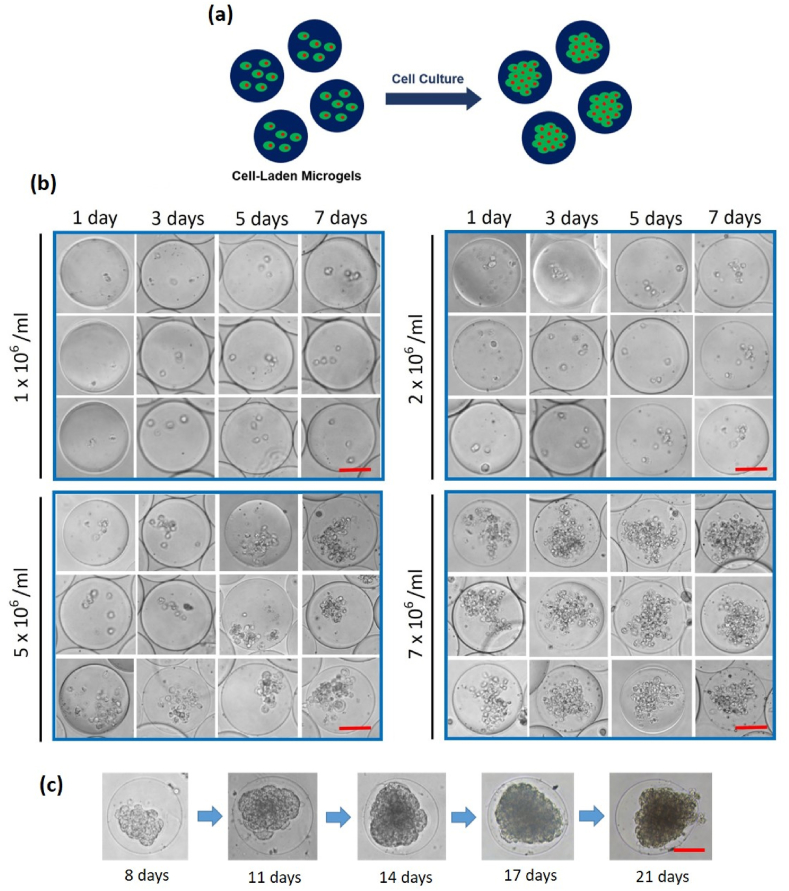
(a) Schematic of cultured cell-laden microgels. (b) Optimization of NK92 cell density, ranging from 1 × 10^6^–7 × 10^6^ cells mL^−1^, to maintain live cell conditions in 3D microgels. (Scale bar: 50 μm) (c) Long term culture of 3D microgels (up to 3 wk). (Scale bar: 50 μm).

The cell growth exhibited by the fabricated microgels over time is shown in Fig. 3A. We screened NK92 cell density to find adoptive cell density to enable maintained viability and proliferation. There were four NK92 cell densities, ranging from 1 × 10^6^ cells mL^−1^ to 7 × 10^6^ cells mL^−1^, maintained for 7 d (Fig. 3B). At 1 × 10^6^ cells mL^−1^, 2 × 10^6^ cells mL^−1^, and 5 × 10^6^ cells mL^−1^ conditions, low cell proliferation was observed. However, 7 × 10^6^ cells mL^−1^ showed high proliferation in microgels. NK92 cells proliferate in a cluster formation in 2D cultures. This result highlights the significance of the density of NK92 cells initially loaded into the microgel. In addition, it was confirmed that the encapsulated NK92 cells could survive adequately when their density was at least 7 × 10^6^ cells mL^−1^. Microgels containing NK92 cells were cultured for a long time (up to 21 d). In contrast, the 2D cultures could be maintained for a few days only, as long-term culturing was difficult due to poor NK92 cell activity. Moreover, we confirmed that the size of the spheroids in NK92 cells increased over time in the microgel environment due to increased cell-to-cell interaction (Fig. 3C).

#### 3.3.2 Effect of microgel mechanics on NK92 cell laden microgels

**Fig. 4:**
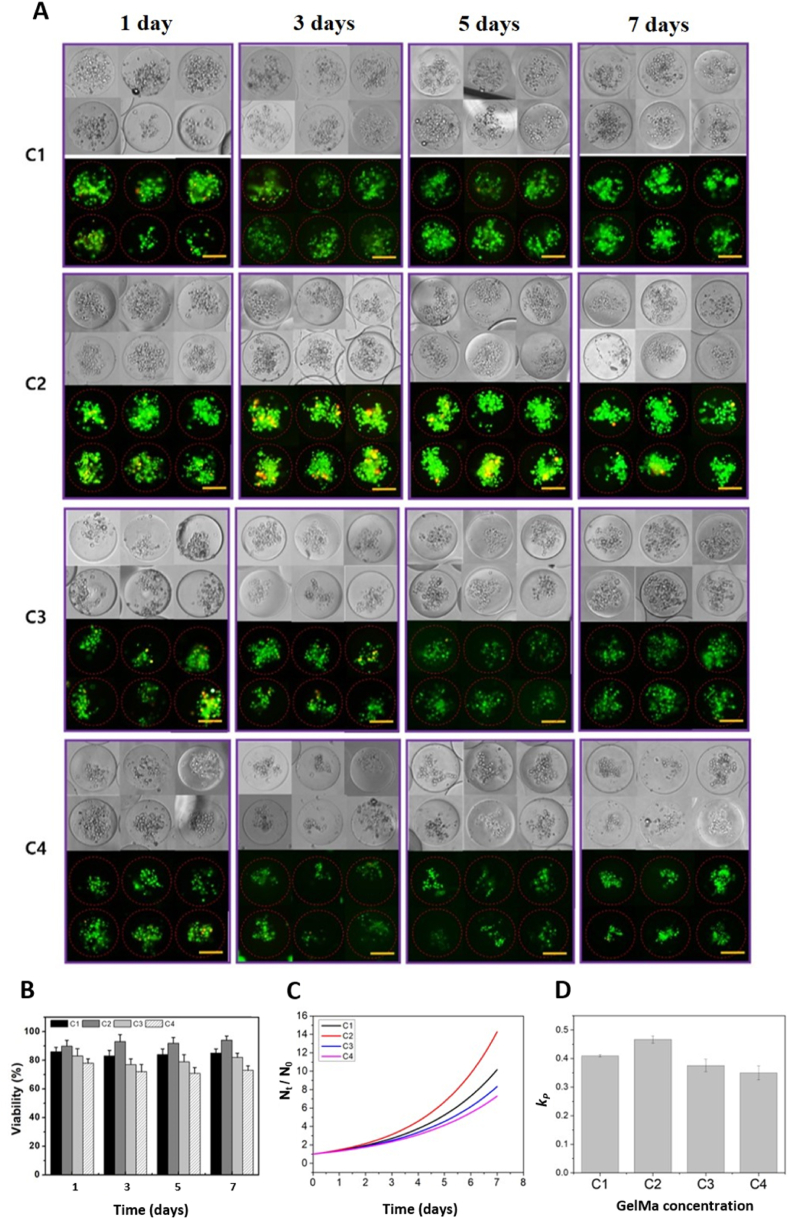
Biocompatibility of NK92 cell-laden microgel for various GelMa concentrations. (a) Images of cell laden microgel LIVE/DEAD assay (C1–C4) up to 7 d. (b) Graphs of cell viability. (c) The normalized number of live cells (N_t_/N_0_) in the microgels was estimated over time (Nt: number of live cells at time, N_0_: number of initial live cells. (d) The plot shown in panel (c) was fitted with a power-law model to obtain the proliferation rates (k_p_).

We used different concentrations of GelMa to optimize NK92 cell proliferation with high viability. The hydrogel stiffness can be controlled with different elastic moduli depending on the polymer concentration. We generated microgels containing NK92 cells with GelMa concentrations ranging from C1 to C4 using a microfluidic chip (Fig. 4A). Fig. 1B and C shows NK92 cell encapsulated droplets changing to microgels because of UV-induced photo-crosslinking. Fig. 2a shows cell-laden droplet formation. Microgels containing NK92 cells maintained at the C2 condition exhibited high proliferation and viability compared to other conditions such as C1, C3, and C4 (Fig. 4B, C, and 4D). C1 was the weakest gel and C4 was the hardest gel. NK92 cells can circulate through blood vessels by adopting lesser (soft) hydrogel stiffness than rigid hydrogels. C2 was the best condition to proliferate and maintain viability. For C3 and C4 conditions, higher GelMa concentrations resulted in greater hydrogel stiffness, which increased crosslinking density, resulting in reduced pore size and lower cell viability due to limited diffusion. The proliferation rate (*k*_p_) was calculated using a power-law model to find the best conditions related to cell proliferation. In our study, C2 condition was the best condition when NK92 cells were encapsulated and cultured for up to 7 d (Fig. 4B, C, and 4D).

### 3.4 NK92 cells cultured in microgel (3D) have higher activity than NK92 cells cultured in 2D

**Fig. 5:**
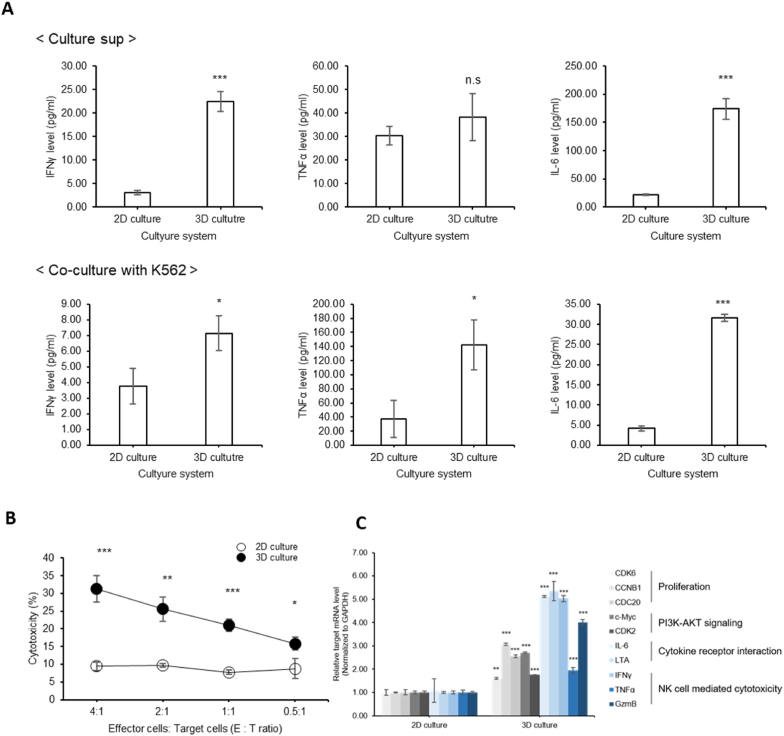
NK92 cells cultured in microgels (3D) have higher activity than NK92 cells cultured in 2D. (a) The levels of IFN-γ, TNF-α, and IL-6 in the NK92 culture medium (upper) or K562 co-cultured medium (bottom) were measured by ELISA. (b) Cytotoxicity test to compare NK92 cells in 2D culture or 3D microgel culture (various E:T ratios). (c) Analysis of specific mRNA expression levels of NK92 cells compared to 2D and 3D culture by RT-PCR. Error bars are ±standard deviation based on technical triplicates. Significance was determined using the Student's t-test ***p < 0.001; **p < 0.01; *p < 0.05.

To distinguish between 2D and 3D cultures, we conducted a comparison of the activity of NK92 cells that were cultured in a 2D or 3D environment. First, we measured the amount of pro-inflammatory cytokine secreted by NK92 cells in the culture medium. The results showed that 3D cultured NK92 cells had higher secretion amounts of interferon-γ (IFN-γ) and interleukin-6 (IL-6) than 2D cultured NK92 cells (Fig. 5A, upper). In addition, we examined cytokines secreted from the medium co-cultured with the K562 target cells. The results showed that more IFNγ, IL-6, and tumor necrosis factor-α (TNF-α) were secreted when co-cultured with 3D cultured NK92 cells (Fig. 5A, bottom). Next, we checked the killing efficacy of NK92 cells cultured in 2D or 3D. Generally, for NK92 cells to react with cancer cells, cell-to-cell interaction is essential. While NK92 cell microgels in 3D cultures exhibited better anti-cancer effects, the surrounding microgel environment can hinder cell-to-cell interactions. Therefore, we dissolved the microgel as ECM structure in order to obtain normal NK92 cell condition. To remove the microgel, we exposed them to collagenase enzyme for 100 min (Fig. S4). Collagenase cannot affect viability of NK92 cells (Fig. S5). After dissolving the microgel by treating with collagenase, we performed a cytotoxicity test with K562 cells. The assessment was conducted at various E/T ratios of 4:1, 2:1, 1:1, and 0.5:1, for 2 h. As shown in the graphs, NK92 cells cultured in 3D microgels showed higher killing efficacy against K562 than NK92 cells cultured in 2D (Fig. 5B). These results indicated that NK92 cells cultured in 3D microgels have enhanced anti-tumor activity compared to 2D cultured NK92 cells. Furthermore, to understand the detailed biological status associated with increased proliferation and activity of 3D cultured NK92 cells, we extracted total RNA and identified the differentially expressed genes. The specific primer sequence is listed in Fig. S6. As a result, we observed that the mRNA level of CDK6, CCNB1, and CDC 20 related to cell growth was increased (Fig. 5C), and several genes associated with PI3K-AKT signaling and cytokine receptor interaction were increased. Altogether, these results indicated that cell proliferation, viability, tumor-killing activity, cytokine production, and NK cell-related gene expression were improved in 3D cultured NK92 cells than in 2D cultured NK92 cells. This is consistent with several reports that when cell-to-cell interaction increases using various 3D culture systems, the viability, proliferation, and activity of NK cells were increased [15,46].

### 3.5 UV power effect of NK92 cell laden microgel and microgel degradation test

**Fig. 6:**
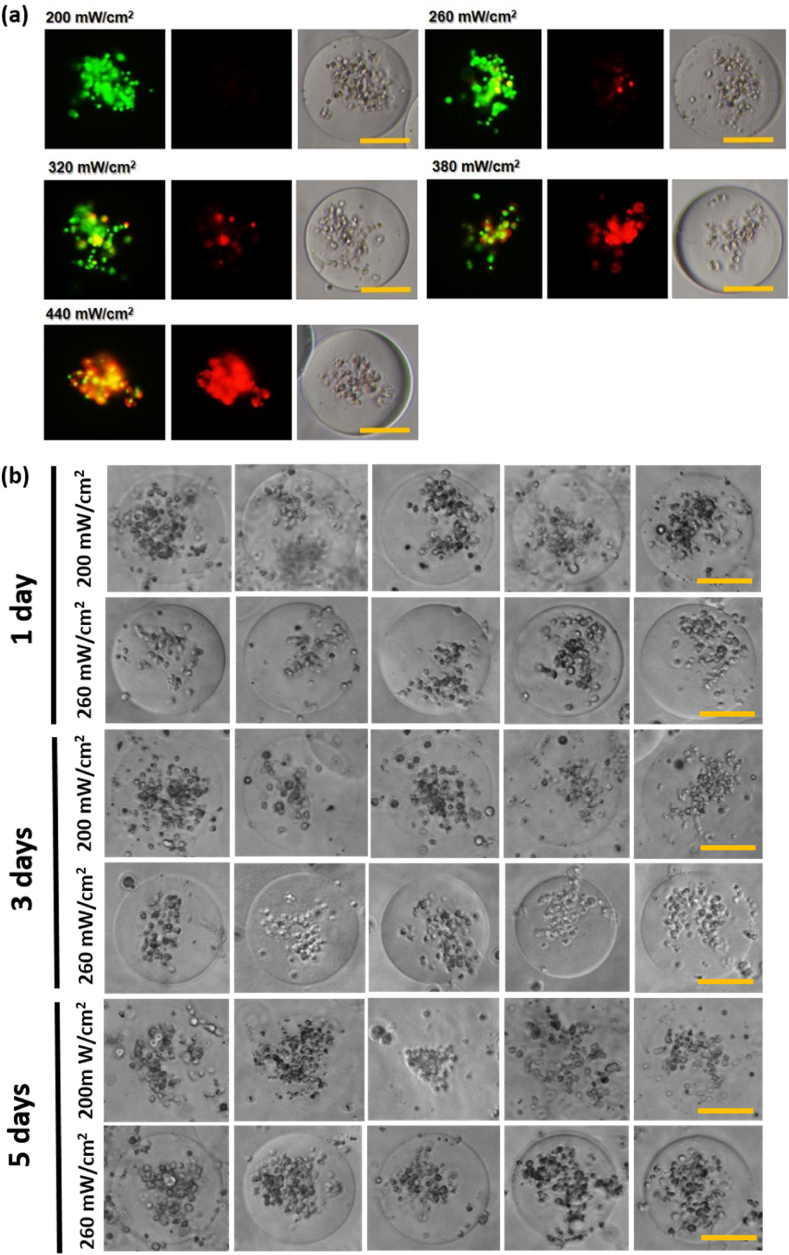
(a) Viability of encapsulated NK92 cells at various UV intensities (0.2–0.44 W cm^−2^). Scale bar: 50 μm (b) Degradation test at Aq1 = 6 %, Aq2 = 9 % (GelMa: C0) conditions by controlling UV intensities 0.2 W cm^−2^ and 0.26 W cm^−2^ (Scale bar: 50 μm).

**Fig. 7:**
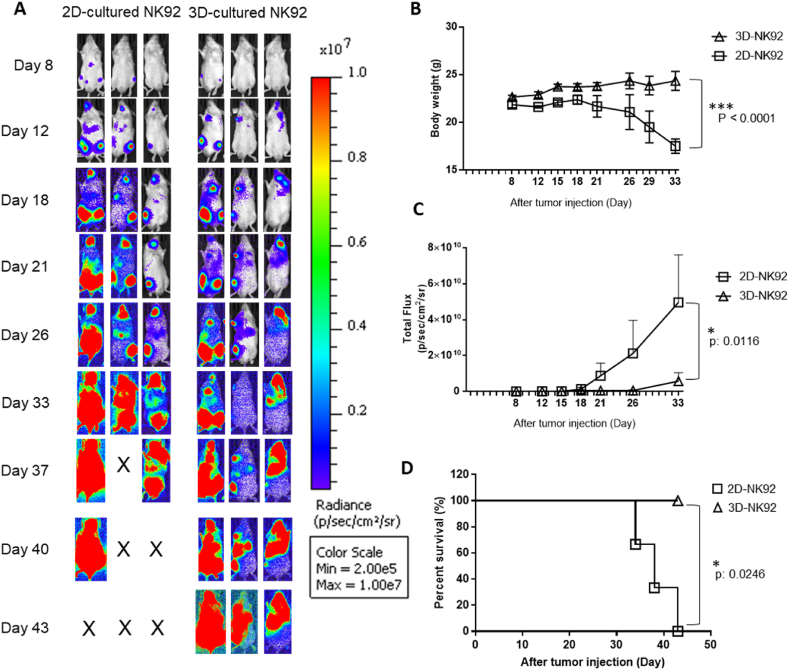
Anticancer effect of micro-bead cultured NK92 cells against K562 leukemia cells. (a) The images of the ventral bioluminescence (BLI) after injection of 2D or micro-bead cultured NK92 cells. (b) Changes in bodyweight were plotted after the injection of K562-Luc cells (n: 3 per group). (c) The values of the ventral BLI were plotted. (d) The survival curves after the injection of K562-Luc cells. Error bars are ± s. e.m. Significance was determined using the two-way ANOVA (A, C) or the Mantel-Cox test (D): ***p < 0.001; **p < 0.01; *p < 0.05.

We conducted an experiment on the viability of NK92 cells according to UV intensity. UV power was used ranging from 0.2 W cm^−2^ to 0.44 W cm^−2^, and was exposed for 1 min (Fig. 6A). As the UV intensity increased, the survival rate of cells started to fall, and it was confirmed that the cells suffered serious damage, especially from 0.38 W cm^−2^ or higher. We made microgels containing NK92 cells by using relatively safe UV intensities of 0.2 W cm^−2^ and 0.26 W cm^−2^. In *in vivo* systems, collagenase D enzyme cannot be used to retrieve NK92 cells. Therefore, we can achieve naturally degradable conditions by changing GelMa concentrations with different UV intensities. At first, we used GelMa 5 % (Aq1) and 8 % (Aq2), in this case, it is not able to maintain to form microgel due to low cross-linking density. GelMa 7 % (Aq1) and 10 % (Aq2), in this case, it is impossible to degrade naturally owing to enough cross-linking for microgel formation. Therefore, at intermediate conditions, for example, GelMa 6 % (Aq1) and 9 % (Aq2), (C0), we fixed this concentration and different UV intensities such as 0.2 W cm^−2^ and 0.26 W cm^−2^ for 1 min (Fig. 6B). For UV exposure at 0.2 W cm^−2^, it is possible to dissolve the microgel; however, 0.26 W cm^−2^ cannot dissolve in culture condition (Fig. 6B). Therefore, GelMa concentrations of 6 % and 9 % were used so that microgels containing NK92 cells could be naturally degraded by cell metabolism. The UV irradiation time was 1 min, and the intensity was 0.2 W cm^−2^. After washing the oil layer, the experiment was carried out under conditions in which all microgels were decomposed within 5 d. By comparing the effects of NK cells cultured in our optimized C2 microgel and NK cells cultured in the microgel (C0) used in Fig. 7 on killing cancer cells, we ensured that there were no problems with the overall flow of the paper. When comparing NK cells cultured in the optimized C2 microgel with C0 microgel manufactured to dissolve naturally, it was confirmed that there was no significant difference in killing power and mRNA expression against cancer cells. However, there was a significant difference between NK cells cultured in 2D and NK cells cultured in microgels. The results were tabulated and attached in Fig. S7.

### 3.6 In vivo antitumor therapy efficiency of the NK92 cell laden microgels

Allogeneic NK cell therapy is one of the potential therapeutic strategies for various cancers in immune cell therapy [47], and among them, adoptive cell transfer is one of the main branches of cancer immunotherapy [48]. Through an *in vitro* assay (Fig. 5), we checked that 3D cultured NK92 cells had increased cell proliferation, viability, tumor-killing activity, cytokine production, and NK cell-related gene expression compared to 2D-cultured NK92 cells. The Microgel system is practical for culture matrix for NK cells and could produce more effective therapeutic outcomes because it is injected in micro-bead units instead of individual cell units during ACT. To test this concept, we identified whether NK92 cells cultured in the microgels would affect tumor-killing efficacy more than 2D cultured NK92 cells. Three mice per group received Luciferase-expressing K562 (K562-Luc) cells, and NK92 cells cultured in 2D systems and NK92 cells cultured into naturally degrade microgels were transfused to immune-deficient mice three times, respectively. We observed that mice injected with 3D cultured NK92 cells had significantly lower bioluminescence signals (Fig. 7A and B) and body weight loss (Fig. 7C) which led to increased survival compared with 2D cultured NK92 cells (Fig. 7D). Therefore, we confirmed that the 3D cultured NK92 cells enhanced the antitumor responses against K562 leukemia more than 2D cultured NK92 cells.

## 4 Discussion

Tumor is one of the important problems for human health. NK cell can detect the target and kill, but it is not good efficiency. In order to enhance NK cell activity, we introduce three-dimensional culture using biocompatible polymer such as Gelatin methacrylate hydrogel. Several research published related to NK cell encapsulated microgel but that has limitation. For example, not homogenous microgel size and difficult to control encapsulated cell density [49]. Furthermore, bulk hydrogel cannot long term cell encapsulated culture due to diffusion limit [15].

Mechanotransduction can induce and determine the differentiation and proliferation of cells by regulating the surrounding environment. Hydrogel can include high water content and can control tunable mechanics through changing hydrogel concentration. In other words, hydrogel degradation rate can control by adaptable concentration. Furthermore, we used GelMa hydrogel, which has biological activity because collagen derived materials and can synthesis double bond from Gelatin to allow UV cross-linking. In order to make microgel formation, we have to use UV light for photo-crosslinking. Generally, UV can damage to viability of the cell but in our study, UV light exposure for 1 min to the encapsulated NK92 cell, this condition cannot critical affect by UV light until UV intensity lower than 0.32 W cm^−2^. With NK92 cell encapsulated microgel by GelMa concentration and NK92 cell prefer C2 condition (Aq1 9 %, Aq2 12 %) compare to soft or hard elastic modulus.

In this study, we introduced the microfluidics-based fabrication of NK92 cell-laden microgels in which tunable mechanics via controlled GelMa concentrations treat tumor immunotherapy both *in vitro* and *in vivo*. Using the microfluidic system, it is possible to generate monodisperse droplets of a gel precursor solution and convert them to microgel formation through UV photo-crosslinking. Microfluidic has a lot of benefit such as mono-disperse in size, easy size control, low sample and reagent consumption. With practical applications in the design of systems in which low volumes of fluids are processed to achieve multiplexing, automation and high-throughput screening. We used the double-flow focusing microfluidic chip method because the core-shell structure could be maintained during droplet generation and the survival rate could be increased because the cells were contained in the core-shell [20,21]. The microgels consisted of biocompatible polymers such as GelMa and can provide a comfortable environment for NK92 cells to proliferate as microgels have a lot of porosity and no diffusion limit. With activated NK cell-laden microgels are designed to completely degrade after 5 days allowing NK cells to be released to achieve more effectively cell-to-cell interaction with cancer cells.

## 5 Conclusions

Compared to 2D-cultured NK92 cells, we checked that the 3D microgel cultured NK92 cells had increased cell proliferation, viability, tumor-killing activity, cytokine production, and NK cell-related gene expression. In addition, through *in vivo* experiments, we confirmed that NK92 cells cultured in 3D microgels can effectively reduce the number of K562 leukemia cells, compared to NK92 cells cultured in 2D. Furthermore, it was found that the animal model using NK92 cells cultured in 3D microgels showed a longer survival rate and the body weight loss and biofluorescence signal were smaller than NK92 cells cultured in 2D. To react between NK92 cell and K562, we have to make degradable microgel design using control UV cross-linking time so that NK92 cell to K562 cell interaction directly. These results suggest that efficient anti-cancer therapy is possible by intravenously injecting microgels containing NK cells, which is much easier than conventional surgical treatment. One of the obstacles to NK cell therapy is the inability to treat solid cancer effectively. We only showed the efficacy of improved NK cell therapy in the leukemia mouse model using K562. Therefore, we have to develop a more advanced 3D microgel system that offers practical therapeutic effects in solid cancer, such as combining antibodies with microgel to enhance tumor targeting or containing an immune adjuvant that can enhance NK cell activity. Taken together, we have demonstrated that the NK cell-laden microgels have great potential as immunotherapy for the treatment of tumors and can be applied to various types of cancers.

## CRediT authorship contribution statement

**Dongjin Lee:** Writing – original draft, Methodology, Investigation, Data curation, Conceptualization. **Seok Min Kim:** Writing – original draft, Methodology, Data curation, Conceptualization. **Dahong Kim:** Formal analysis. **Seung Yeop Baek:** Methodology, Formal analysis, Data curation. **Seon Ju Yeo:** Investigation, Formal analysis. **Jae Jong Lee:** Investigation. **Chaenyung Cha:** Investigation. **Su A Park:** Writing – review & editing, Validation, Supervision, Project administration, Funding acquisition. **Tae-Don Kim:** Writing – review & editing, Validation, Supervision, Project administration, Funding acquisition, Conceptualization.

## Declaration of competing interest

The authors declare that they have no known competing financial interests or personal relationships that could have appeared to influence the work reported in this paper.

## Data Availability

No data was used for the research described in the article.

## References

[1] Bray F. (2018). CA A Cancer J. Clin..

[2] Chiossone L. (2018). Nat. Rev. Immunol..

[3] Mellman I. (2011). Nature.

[4] Wang W. (2020). Cancer Lett..

[5] (2019). EBioMedicine.

[6] Chow V.A. (2019). Am. J. Hematol..

[7] Paul S., Lal G. (2017). Front. Immunol..

[8] Chan C.J. (2014). Cell Death Differ..

[9] Vivier E. (2008). Nat. Immunol..

[10] Kim J.Y. (2006). Exp. Mol. Med..

[11] Deng W. (2015). Science.

[12] Rezvani K. (2017). Mol. Ther..

[13] Maki G. (2003). Bone Marrow Transplant..

[14] Liu S. (2021). J. Hematol. Oncol..

[15] Ahn Y.H. (2020). Biomaterials.

[16] Wang C. (2009). Biomaterials.

[17] Guo M.T. (2012). Lab Chip.

[18] Teh S.Y. (2008). Lab Chip.

[19] Schneider T. (2013). Anal. Chem..

[20] Kim S. (2016). Colloids Surf. B Biointerfaces.

[21] Aikawa T. (2012). Langmuir.

[22] Aikawa T. (2013). Soft Matter.

[23] Rossow T. (2012). J. Am. Chem. Soc..

[24] Cha C. (2014). Biomacromolecules.

[25] Zhao X. (2016). Adv. Funct. Mater..

[26] Park K.-S. (2016). Macromol. Res..

[27] Chen Y.C. (2012). Adv. Funct. Mater..

[28] Bertassoni L.E. (2014). Lab Chip.

[29] Hasan A. (2015). Biomed. Microdevices.

[30] Levett P.A. (2014). Acta Biomater..

[31] Fu F. (2016). ACS Appl. Mater. Interfaces.

[32] Topkaya S.N. (2015). Biosens. Bioelectron..

[33] Puckert C. (2017). Soft Matter.

[34] Yue K. (2015). Biomaterials.

[35] Nichol J.W. (2010). Biomaterials.

[36] Yue K. (2017). Biomaterials.

[37] Haycock J.W. (2011). Methods Mol. Biol..

[38] Ravi M. (2015). J. Cell. Physiol..

[39] Thoma C.R. (2014). Adv. Drug Deliv. Rev..

[40] van Duinen V. (2015). Curr. Opin. Biotechnol..

[41] Kim S. (2017). Macromol. Biosci..

[42] Jang J., Cha C. (2018). Biomacromolecules.

[43] Lee D. (2018). Advanced Biosystems.

[44] Chu C. (2009). Tissue Eng..

[45] Sikkema J. (1995). Microbiol. Rev..

[46] Kim T.J. (2014). Sci. Rep..

[47] Geller M.A., Miller J.S. (2011). Immunotherapy.

[48] Laskowski T.J. (2022). Nat. Rev. Cancer.

[49] Wu D. (2019). ACS Appl. Mater. Interfaces.

