# Supplementary material for: Microfluidics-assisted fabrication of natural killer cell-laden microgel enhances the therapeutic efficacy for tumor immunotherapy
Source: Mater Today Bio. 2024 Apr 17;26:101055. doi: 10.1016/j.mtbio.2024.101055 (PMC11061753; doi:10.1016/j.mtbio.2024.101055)
Supplement: Supplementary file 1 [file mmc1.docx]

**Supporting Information**

Microfluidics-assisted fabrication of natural killer cell-laden microgel enhances the therapeutic efficacy for tumor immunotherapy

Dongjin Lee^a,b†^, Seok Min Kim^a†^, Dahong Kim^b,d^, Seung Yeop Baek^e^, Seon Ju Yeo^b^, Jae Jong Lee^b^, Chaenyung Cha^e,f^, Su A Park^b*^ and Tae-Don Kim^a,c*^

*Corresponding author. Email: [psa@kimm.re.kr](mailto:psa@kimm.re.kr) (S.A.P.); [tdkim@kribb.re.kr](mailto:tdkim@kribb.re.kr) (T.D.K.)


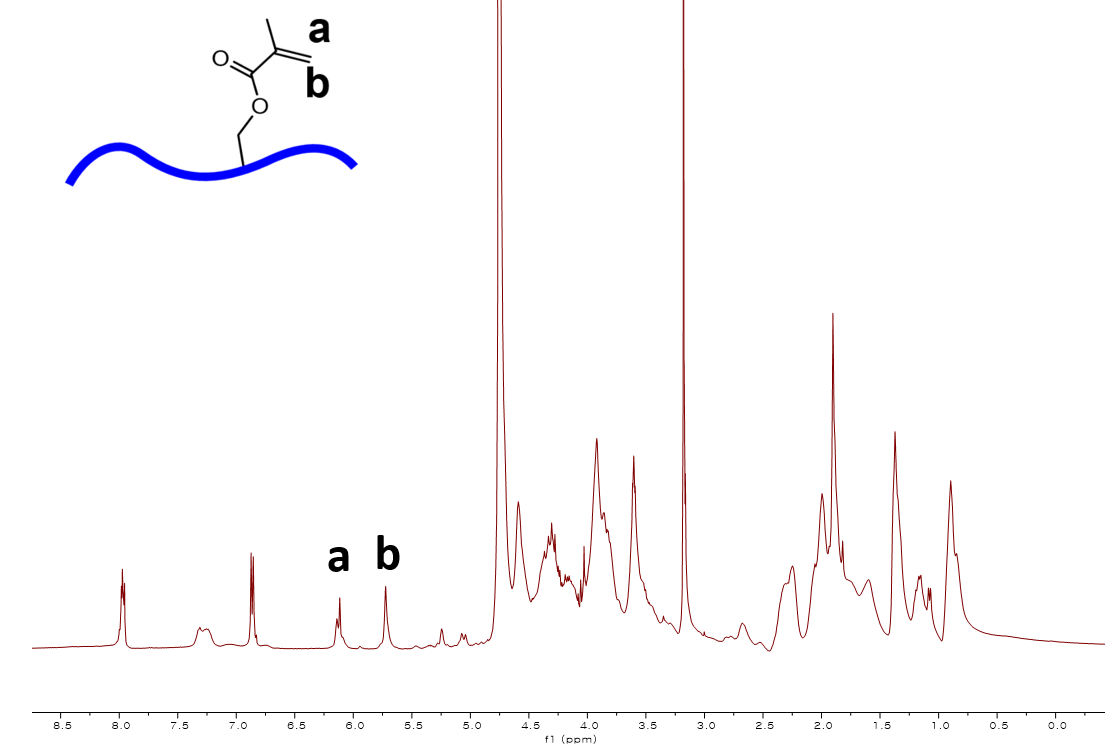


**Fig. S1.** ^1^H-NMR spectra of methacrylic gelatin hydrogel. (a and b) The two kinds of methacrylic peaks are shown.

**
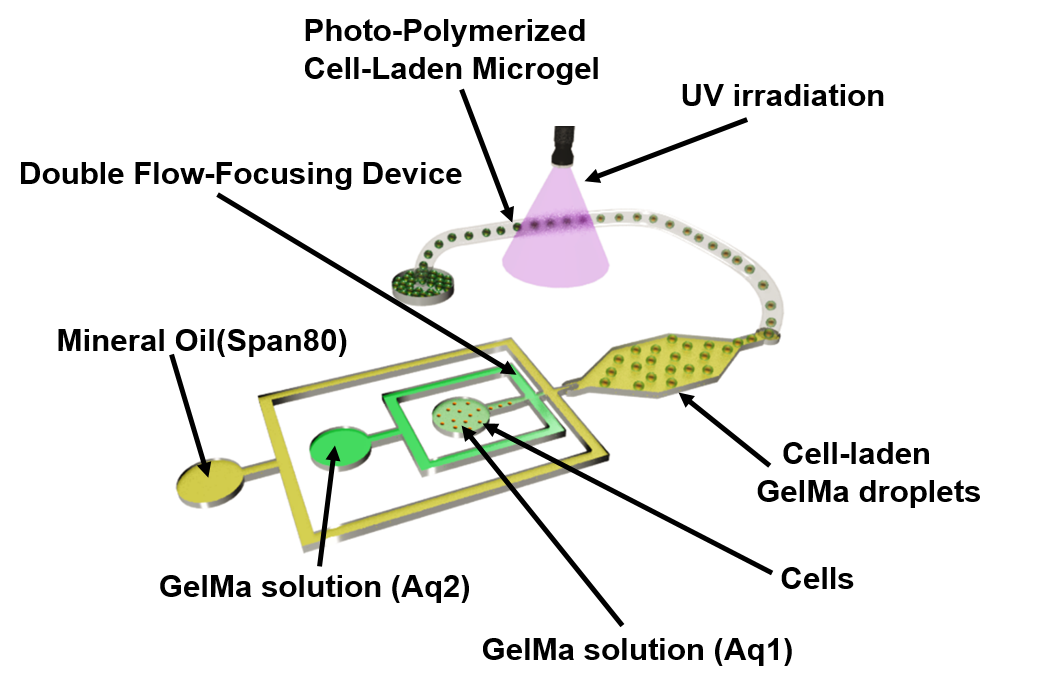
**

**Fig. S2.** Overall experimental schematic of the double flow-focusing microfluidic chip used to generate cell encapsulated microgels.

**
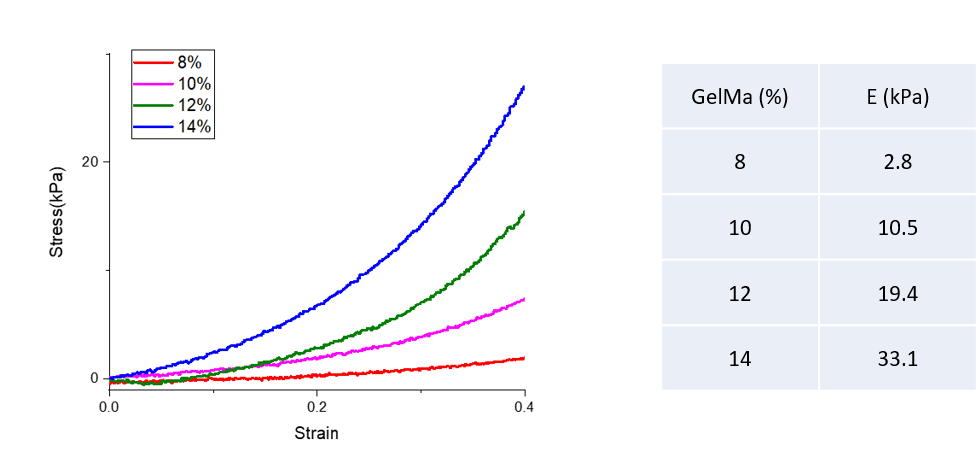
**

**Fig. S3.** Stress-strain curves of methacrylic gelatin hydrogels at different concentrations gained by uniaxial compression. The table shows the average elastic moduli (E) of methacrylic gelatin hydrogels calculated from the curves.


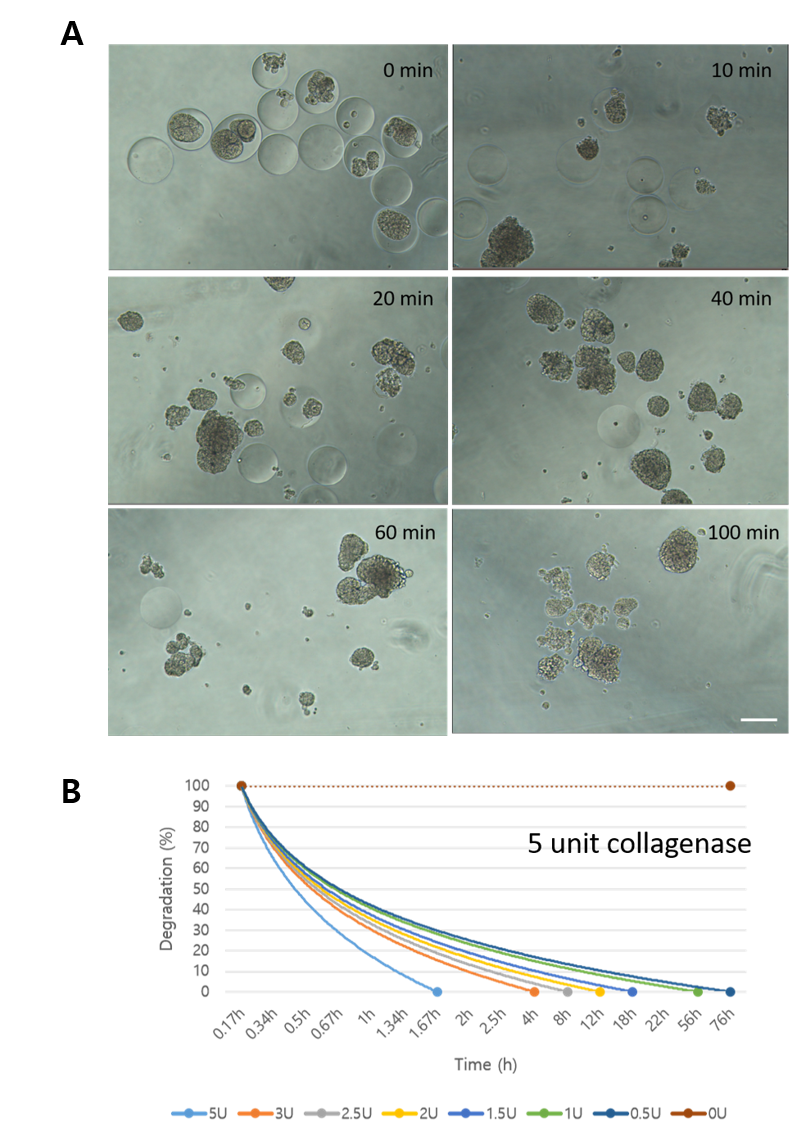


**Fig. S4.** A. Images of cell encapsulated microgels which were removed by the collagenase enzyme over time. B. Graphs of degradation time versus collagenase enzyme concentration.


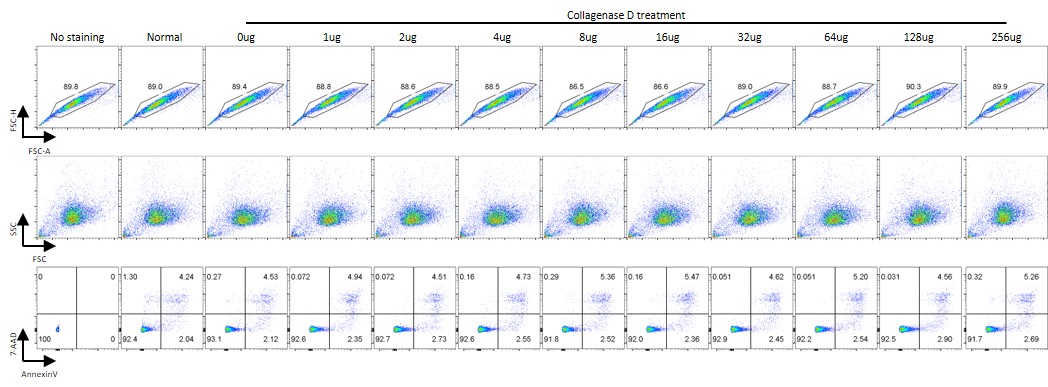


**Fig. S5.** NK92 cell viability under the activity of collagenase concentration ranging from 0 µg to 256 µg.


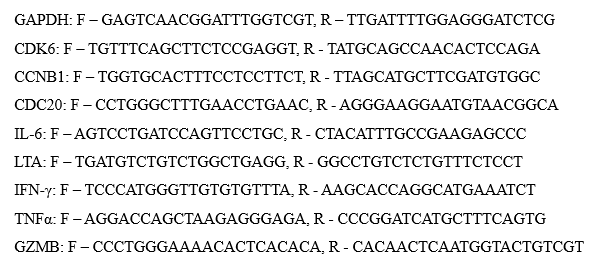


**Fig. S6.** The specific primer sequence list.


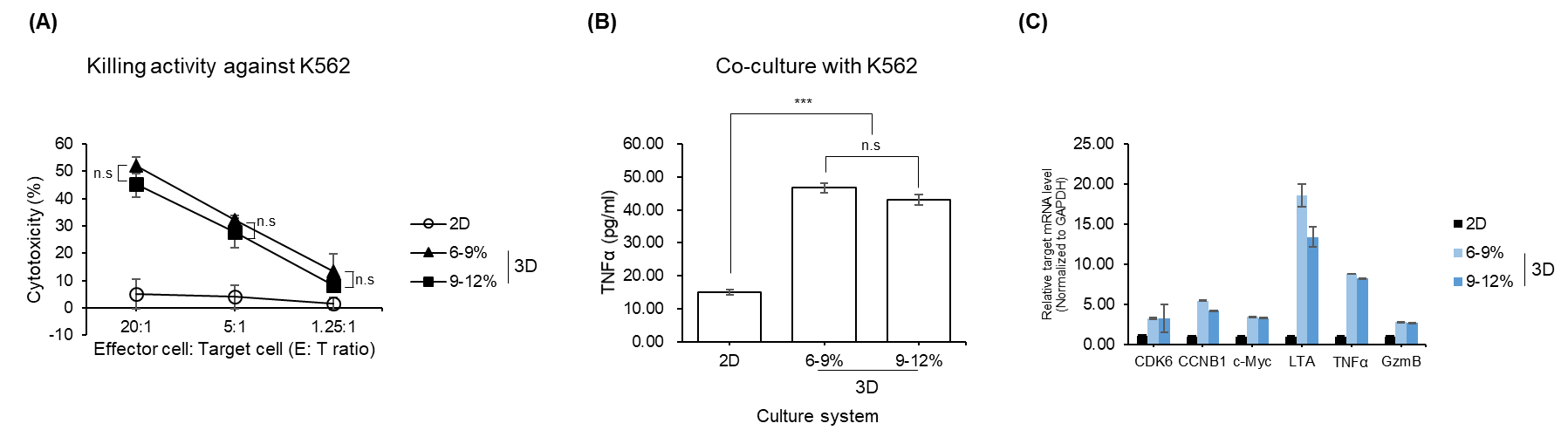


**Fig. S7.** No change in the activity of NK cells according to the composition of the microgel used as a 3D culture system.(A) K562 cells killing activity by 2D or 3D cultured (6-9% or 9-12%) NK92 cells was measured by a calcein AM-based cytotoxicity assay at the indicated NK92: K562 (E: T) ratio. (B) Levels of TNFα in the co-culture media with K562 cells for 4 hours were determined by ELSIA. (C) The expression levels of specific mRNA associated with activity in NK92 cells cultured in 2D or 3D (6-9% or 9-12%) were compared by RT-qPCR. Error bars are ± s.d. based on three (A, B) or two (C) technical replicates. Significance was determined using the or Student’s t-tests (A, B): ***p < 0.001; **p < 0.01; *p < 0.05.
